# Pilose Antler Extracts (PAEs) Protect against Neurodegeneration in 6-OHDA-Induced Parkinson's Disease Rat Models

**DOI:** 10.1155/2019/7276407

**Published:** 2019-01-08

**Authors:** Chaohua Li, Yanan Sun, Weifeng Yang, Shuhua Ma, Lili Zhang, Jing Zhao, Xin Zhao, Yi Wang

**Affiliations:** ^1^Xiyuan Hospital, China Academy of Chinese Medical Sciences, No. 1 Xiyuan Playground, Haidian District, Beijing 100091, China; ^2^Experimental Research Center, China Academy of Chinese Medical Sciences, No. 16, Nanxiao Road, Dongzhimen, Beijing 100700, China; ^3^School of Traditional Chinese Medicine, Shenyang Pharmaceutical University, 103 Wenhua Road, Shenhe District, Shenyang, Liaoning Province 110016, China; ^4^School of Chinese Medicinal Materials, Jilin Agricultural University, No. 2888 Xincheng Avenue, Changchun City, Jilin Province 130118, China

## Abstract

Parkinson's disease (PD) is one of the most common neurodegenerative diseases worldwide. Although dopamine replacement therapy mitigates motor dysfunction in PD patients, there are no therapeutics that are currently available to reverse neuronal cell death in the substantia nigra pars compacta (SNc), which is the main region for dopamine loss in PD patients. The protein concentration of the Pilose antler extracts (PAEs) was estimated using the Bradford Protein Assay Kit. Hematoxylin and eosin (HE) staining was used to evaluate the protective effect of PAEs on 6-OHDA induced cell death in PD model rats. Immunohistochemistry (IHC) was used to detect the tyrosine hydroxylase (TH) positive neuronal cell in SNc. HPLC-MS was used to detect dopamine (DA), 3,4-Dihydroxyphenylacetic acid (DOPAC), homovanillic acid (HVA), 5-hydroxytryptamine (5-HT), 5-hydroxyindoleacetic acid (5-HIAA), and glutamate (Glu) levels in the striatum and cerebrospinal fluid (CSF). The amino acid level in the striatum and CSF was measured by HPLC-FLD. Protein expression of growth associated protein-43 (GAP-43) and neurofilament heavy polypeptide (NF-H) was measured using western blotting. The components of PAEs through blood vessels were detected by HPLC/MS/MS. In this study, PAEs with proteins ranging from 10 kDa to 250 kDa molecular weight was administered to 6-OHDA-induced PD rats. We found that PAEs inhibited 6-OHDA-induced neuronal cell death and TH-positive neuronal loss in SNc. PAEs administration also increased the levels of DA, DOPAC, and 5-HT, in addition to DOPAC/DA and HVA/DA indexes in the CSF and Striatum of 6-OHDA induced rats. Conversely, PAEs decreased the levels of Glu and GABA. Treatment with PAEs and Madopar increased GAP-43 and NF-H expression in the SNc and striatum. Proteomic analysis using LC/MS/MS indicated that 11 components of PAEs may have neuropharmacological effects. These results demonstrate that PAEs protects against 6-OHDA induced toxic effects in the PD rat models. Intragastric administration of PAEs may be a novel therapeutic strategy for neurodegenerative disorders like PD.

## 1. Introduction

PD is the second most common neurodegenerative disease worldwide and affects approximately 1-2% of the population over 60 years of age. The pathological characteristics of PD are progressive loss of dopaminergic (DAergic) neurons in the SNc and the accumulation of intracellular proteinaceous aggregates termed Lewy bodies. These result in motor and nonmotor disorders, including resting tremor, bradykinesia, muscular rigidity and gait disturbance, depression, sleep disturbances, and anosmia [1, 2].

Current approved therapeutics attenuate the symptoms of PD but do not prevent the degeneration of DAergic neuronal cells and hence fail to reverse disease progression. These drugs have serious adverse effects after long-term administration and may even exacerbate PD progression [3–5]. Thus, more efficacious and safer drugs to treat PD are required.

Pilose antler (PA, Lu-Rong in Chinese), especially PA from the sika deer (*Cervus Nippon Temminck*) produced in Jilin Province of China, is a famous Traditional Chinese Medicine (TCM). It has abundant active components such as mineral elements, amino acids, peptides, proteins, polysaccharides, fatty acids, and phospholipids. Of these components, proteins and peptides are the critical components and constitute approximately 55.26% of PA [6].

Traditionally, PA is used as a Chinese tonic in TCM practice. Over the recent years, it has been reported that PA and its components have neuroprotective effects* in vitro*. However, supporting evidence is limited to* in vitro *studies or studies performed using local injection administration. This route of administration is not consistent with PA intragastric administration. In addition, our previous study demonstrated that the PA protein constituents after intragastric administration underwent complex structural changes. This finding made it imperative to study the pharmacological function of PA using* in vivo* methods. However, blood serum makes it difficult to analyze proteins or peptides using current available methods. Molecular tracing techniques using fluorescent probes provide a new approach to analyze protein or peptide components absorbed into the bloodstream.

In our previous study we demonstrated that certain protein and peptide constituents in PA were absorbed into the bloodstream through the gastric mucosa [7]. The data suggested that the pharmacological function of the PA components were likely to function as proteins or peptides that originated from intragastric administrated PAEs. In this study, we investigated the neuroprotective effects of intragastric administrated PAEs using the 6-OHDA-induced PD rat models. Using fluorescent tracing techniques and proteomic methods, the active ingredients of PAEs were analyzed to determine the neuroprotective mechanism of PA.

## 2. Methods

### 2.1. Materials

Materials used were 6-hydroxydopamine hydrobromide (6-OHDA, Sigma, USA), apomorphine (National Institutes for Food and Drug Control, China), Madopar (Shanghai Roche Pharmaceuticals Ltd., China), paraformaldehyde perfusion (PFA, Solarbio, China), hematoxylin and eosin (HE, Beijing Berlin Biological Co., Ltd., China), rabbit anti-tyrosine hydroxylase (TH) n-terminal polyclonal antibody (Sigma, USA), Polink-2 plus® Polymer HRP Detection System (PV-9001, ZSGB-BIO, China), and DAB (MXB Co., Ltd., China)

### 2.2. Preparation of PAEs

Fresh PA was obtained from a local deer farm (Jiyunluye Ltd., China). PAEs were extracted using acetic acid as follows: first, PA was micronized using a XDW-6B Low temperature superfine mill (Beijing KunjieYucheng machine Co., Ltd., China). Acetic acid was then added to maintain the pH at 4.0. The mixture was then centrifuged at 5,000 g for 15 min at 4°C, and the supernatant was extracted and placed in the R-215 Rotavator (Buchi, Switzerland) at 55°C. The concentrated supernatant was then dialyzed using a P1000D dialysis membrane (Solarbio, China) and lyophilized using a FD-1D-50 freeze drying machine (Beijing Boyikang laboratory instruments Medical Co., Ltd., China). The PAEs powder was stored at 4°C until required. The protein concentration of the PAEs was estimated using the Bradford Protein Assay Kit (Beyotime, China).

### 2.3. Estimating Molecular Weight of PAEs Components by SDS-PAGE

Sodium dodecyl sulphate polyacrylamide gel electrophoresis (SDS-PAGE) performed the molecular weight of the PAEs components. 20 *μ*l (10 mg/ml) of protein samples was loaded into each lane and run at a constant voltage of 120 V. The gel was stained using Coomassie Brilliant Blue and the resulting protein bands were quantified with Photoshop.

### 2.4. 6-Hydroxydopamine-Induced and PAEs Administration

All animal experiments should be carried out the National Institutes of Health Guide for the Care and Use of Laboratory Animals. We make all efforts to minimize animal suffering and reduce the number of animals used. Eighty adult male Wistar rats (Vital River Laboratories, China) weighing 190-210 g each were used in this study. All animals were group housed, with free access to food and water in a climate-controlled room. Rats were immobilized using a stereotaxic instrument (RWD life science in Shenzhen, China) and received a single injection of 6-OHDA (8 *μ*g, 2 *μ*l /min) to the right brain (A: -4.8 mm, L: + 2.0 mm, H: 8.0 mm from the substantia nigra (SN) and A: -4.8 mm, L: +1.2 mm, H: 8 mm from the ventral tegmental area (VTA)). After three weeks, animals were injected with apomorphine intraperitoneally (0.5 mg/kg). Rotational asymmetry toward the lesion side was recorded and measured. A successful PD rat model was established if rats had a rotation circle number > 210 r/h. PD rats were divided into 5 groups: the control group (C), the model group (M), PAEs-Low dose group (PAEs-L), PAEs-High dose group (PAEs-H), and the positive control group (Madopar), with 5 PD rats in each group. For PAEs-L and PAEs-H, rats were intragastric administrated with 60 mg/kg·d and 180 mg/kg·d PAEs for 14 days, respectively. The Madopar group received intragastric administration of Madopar (10 mg/kg·d) for 14 days. The control group received an equal volume of saline.

### 2.5. LC-MS Analysis of Neurotransmitters in Brain Tissue and Cerebrospinal Fluid (CSF)

After the animals were sacrificed, the nigra and striatum were harvested. After blotting to remove excess moisture, the samples were weighted, recorded, and stored at -80°C until analyzed. For neurotransmitter detection, 5 ml (water)/g (sample) was used for homogenization and then centrifuged at 5000 rpm for 30 min at 4°C. 60 *μ*l of the supernatant was collected and 180 *μ*l acetonitrile was added to the supernatant. Next, 10 *μ*l 0.2 ng/ ml d-DA and d-5HT, which served as deuterium internal standards, was added to the supernatant, followed by centrifugation at 10000 g for 10 min at 4°C. 195 *μ*l supernatant was then collected and dried using nitrogen gas. The dried material was then dissolved in 45 *μ*l 0.1% formic acid. Changes in 5-hydroxytryptamine (5-HT), dopamine (DA), adrenaline (AD) and noradrenaline (NE), and dihydroxyphenylacetic acid (DOPAC) levels after PAEs treatment in brain tissue and CSF were then measured by LC-MS analysis.

### 2.6. High-Performance Liquid Chromatography with Fluorescence Detector (HPLC-FLD) for Amino Acid Measurements

Acetonitrile was added to the homogenized nigra and striatum supernatants at a ratio of 1:2. The samples were then centrifuged at 12000* g* for 10 min at 4°C twice. The supernatant was collected and diluted with water at a ratio of 1:9. Amino acids in the samples were detected using HPLC-FLD. Chromatography conditions for HPLC-FLD were as follows: injection volume was 15 *µ*l; the mobile phase A was 20 mM sodium acetate/methanol/tetrahydrofuran (400/95/5,* v*/*v*/*v*); phase B was sodium acetate/methanol (120/380,* v*/*v*); flow rate was 0.8 ml/min; fluorescence detection was performed at an excitation wavelength of 340 nm and an emission wavelength of 450 nm.

### 2.7. Hematoxylin and Eosin Staining

After anesthesia, rats were perfused intracardially with 0.9% saline, followed by 4% PFA for 24 h. After 4% PFA fixation and paraffin embedding, 6 *µ*m thick slices was obtained from each section and was stained with HE. Neuron morphology was observed using the BX61 microscope (Olympus, Japan) at 100 x magnification.

### 2.8. Tyrosine Hydroxylase Immunohistochemistry

Tissue slices underwent microwave antigen retrieval in citric acid buffer (PH 6.0) for 20 min. Slices were then permeabilized and washed in a solution of 0.025% Triton X-100 in 0.01 M TBS two times for 5 min at room temperature. To block unspecific binding sites, slices were incubated with normal goat serum for 2 h at room temperature. Subsequently, the slices were incubated with a rabbit anti-tyrosine hydroxylase polyclonal antibody (1:100) at 4°C overnight. The slices were then washed with TBS two times and incubated with Polink-2 plus® Polymer HRP Detection System (1:1000) for 60 min at room temperature. Tissues were developed using DAB. Nigral TH-positive neurons were observed using the BX61 microscope (Olympus, Japan) at 40 x magnification.

### 2.9. Western Blotting

Protein expression of growth associated protein-43 (GAP-43) and neurofilament heavy polypeptide (NF-H) was measured using western blotting. Proteins were resolved in 10% polyacrylamide gels, transferred to Polyvinylidene fluoride (PVDF) membranes and incubated with a polyclonal antibody against GAP-43 or NF-H at 4ºC overnight. Subsequently, the membranes were washed and incubated in their respective secondary antibodies (1:1000) for 2 h at room temperature. Protein detection was performed using the enhanced chemiluminescence kit (Amersham Imager 600, GE, USA). The intensity of protein bands was determined by normalizing against the intensity of GAPDH. The relative band intensity ratio of the treated group over the control group was then calculated.

### 2.10. LC-MS-MS Identification of PAEs Components after Intragastric Administration

Proteins were separated on SDS-PAGE gels. The gels were then horizontally cut into 10 slices. The slices were then processed for LC/MS/MS.

#### 2.10.1. SDS-PAGE Gel Slice Pretreatment

Gels were dissolved in DTT at 56°C, and then decolorized after iodoacetamide alkylation reaction. The gels were then hydrolyzed with trypsin after vacuum drying at 37°C for 15 h.

#### 2.10.2. Instrument Parameter Setting

Chromatography was performed using a C18 column. The mobile phase consisted of water with 0.1% formic acid (phase A) and acetonitrile with 0.1% formic acid (phase B). The mobile phase was eluted gradiently with 5%-25% phase B at 5-70 min; 25%-35% phase B at 70-80 min; 35%-80% phase B at 80-90 min; 80%-80% phase B at 90-100 min. The flow rate was 400 *μ*l/min.

MS conditions were as follows: spray voltage, 2000 V; ion transfer tube temperature, 320°C; MS1 detector, Orbitrap; MS1 resolution, 120,000; MS1 scan range, 350–1550 m/z; MS2:HCD, collision energy, 36%; MS2 detector, ion trap.

### 2.11. Statistical Analysis

Statistical analysis was performed using SPSS version 16.0 program (SPSS, Inc., USA). Data was expressed as mean ± standard deviation (SD). Differences among all groups were analyzed by one-way ANOVA test. P< 0.05 was considered statistically significant.

## 3. Results

### 3.1. Identification of PAEs Components

**Figure 1:**
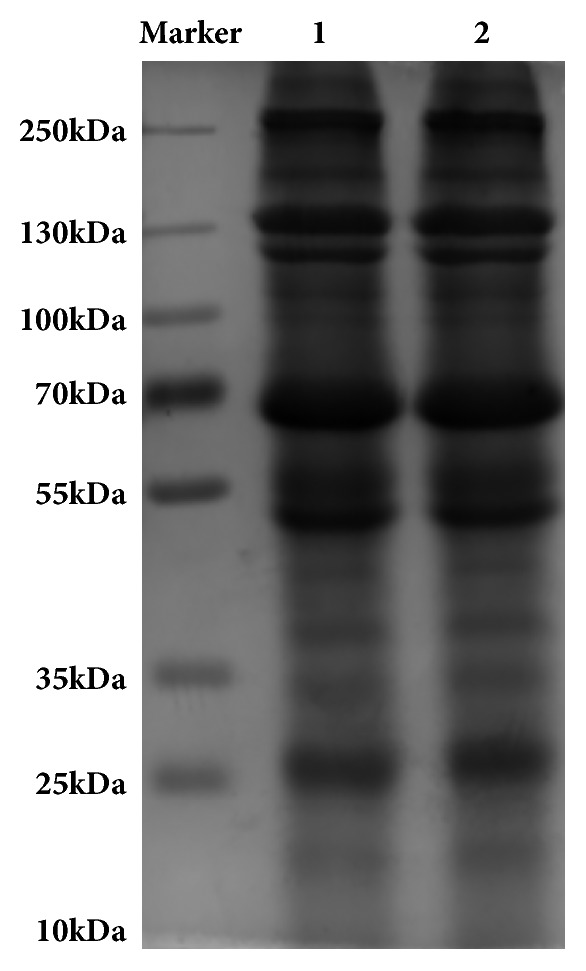
Molecular weight profile analysis of PAEs using SDS-PAGE. Protein components of PAEs mainly ranged from 25 kDa to 70 kDa. M: marker; 1, 2: PAEs samples.

The protein content of the PAEs was 36.69%. The molecular weight of the proteins and peptides in PAEs ranged between 10 to 250 kDa, with the majority of them between 25 kDa to 70 kDa (Figure 1).

### 3.2. Effect of PAEs on Monoamine Content in CSF

**Figure 2:**
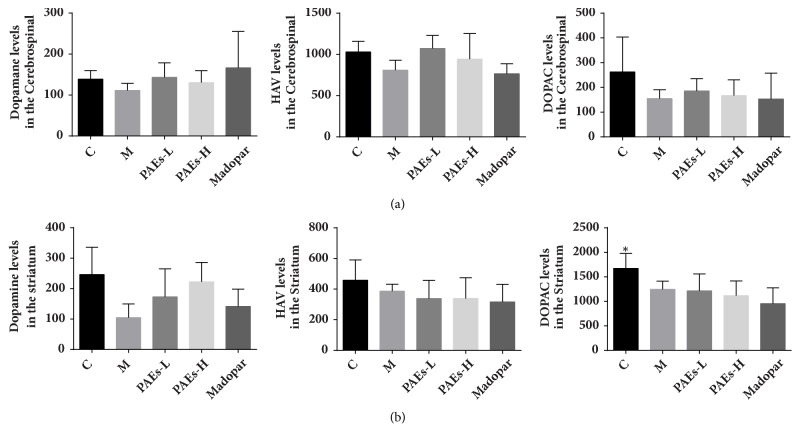
PAEs upregulated the levels of DA and its metabolites in CSF and striatum of PD rats model. HPLC-MS was used to measure DA, DOPAC, and HAV levels. (a) 6-OHDA decreased DA, HAV, and DOPAC levels in CSF of rats, while 60 mg/kg·d and 180 mg/kg·d PAEs administration increased DA, HAV, and DOPAC levels in CSF, but not significant. (b) 6-OHDA decreased DA, HAV and DOPAC levels in striatum of rats, while 60 mg/kg·d and 180 mg/kg·d PAEs administration increased DA, HAV, and DOPAC levels in striatum, but not significant (*∗*p<0.05 versus model, *∗∗*p<0.01 versus model).

Monoamines in CSF were determined using HPLC-FLD. The DA contents and its metabolites (DOPAC and HAV) in the striatum decreased in response to 6-OHDA lesion formation, while administration of PAEs-L and PAEs-H increased the DA contents and its metabolites in the CSF of rats with 6-OHDA lesions; however this increase was not significant. Administration of Madopar increased only the DA contents in CSF (Figure 2(a)).

**Figure 3:**
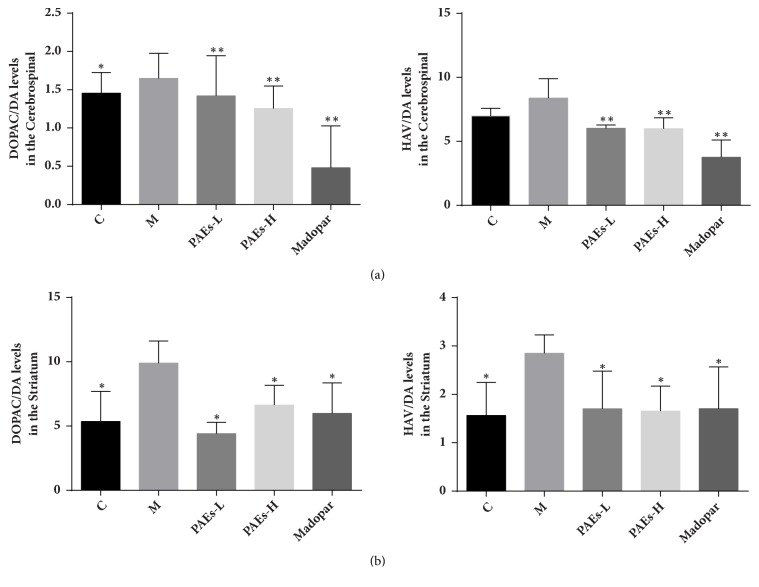
PAEs upregulated the levels of DOPAC/DA and HAV/DA in CSF and striatum of PD rats model. (a) 6-OHDA increased DOPAC/DA and HAV/DA in CSF of rats, while 60 mg/kg·d and 180 mg/kg·d PAEs administration decreased DOPAC/DA and HAV/DA in CSF. (b) 6-OHDA increased DOPAC/DA and HAV/DA in striatum of rats, while 60 mg/kg·d and 180 mg/kg·d PAEs administration significantly decreased DOPAC/DA and HAV/DA in striatum (*∗*p<0.05 versus model, *∗∗*p<0.01 versus model).

Compared to the control group, the DOPAC/DA levels in the model group increased significantly. However, administration of PAEs or Madopar significantly decreased DOPAC/DA and HAV/DA levels (*P*<0.01) (Figure 3(a)).

**Figure 4:**
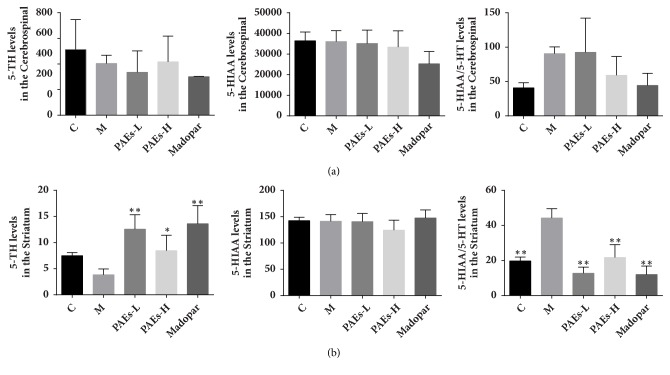
PAEs upregulated the levels of 5-HT, 5-HIAA and 5-HT/5-HIAA in CSF and striatum of PD rat model. HPLC-MS was used to measure 5-HT and 5-HIAA levels. (a) 6-OHDA decreased 5-HT level, and increased 5-HT/5-HIAA in CSF of rats, while 180 mg/kg·d PAEs administration increased 5-HT level, and decreased 5-HT/5-HIAA in CSF. (b) 6-OHDA decreased 5-HT level, and increased 5-HT/5-HIAA in striatum of rats, while 60 mg/kg·d and 180 mg/kg·d PAEs administration significantly increased 5-HT level, and also decreased 5-HT/5-HIAA in striatum (*∗*p<0.05 versus model, *∗∗*p<0.01 versus model).

In the model group, 5-HT levels decreased with no significant changes to 5-HIAA levels. The 5-HIAA/5-HT ratio was not significantly different between the model and control group (*P* > 0.05). After PAEs and Madopar administration, the 5-HIAA and 5-HT levels and 5-HIAA/5-HT ratio increased but were not statistically significant compared to the control group (Figure 4(a)).

### 3.3. Effect of PAEs on Content of Monoamines in the Brain

Monoamines in the striatum were measured using HPLC-MS. Compared to the control group, 58% reduction in DA and 26% reduction in DOPAC levels were observed. In the model group, HAV levels were also decreased but not significantly compared to the control group. After PAEs-H administration, DA levels increased to similar levels as the control. DA levels in the PAEs-H group were also higher compared to the Madopar group. PAEs administration had no influence on DOPAC and HAV levels (Figure 2(b)).

DOPAC/DA levels in the model group were significantly increased compared to the control group. After PAEs and Madopar administration; DOPAC/DA and HAV/DA ratios were significantly decreased (P<0.01) (Figure 3(b)).

In the model group, 5-HT levels were reduced while 5-HIAA levels remained unchanged (P>0.05). However the 5-HIAA/5-HT ratio was remarkably increased. After PAEs and Madopar administration, the 5-HT levels increased while the 5-HIAA/5-HT ratio decreased (Figure 4(b)).

### 3.4. Effect of PAEs on Amino Acid Content

**Figure 5:**
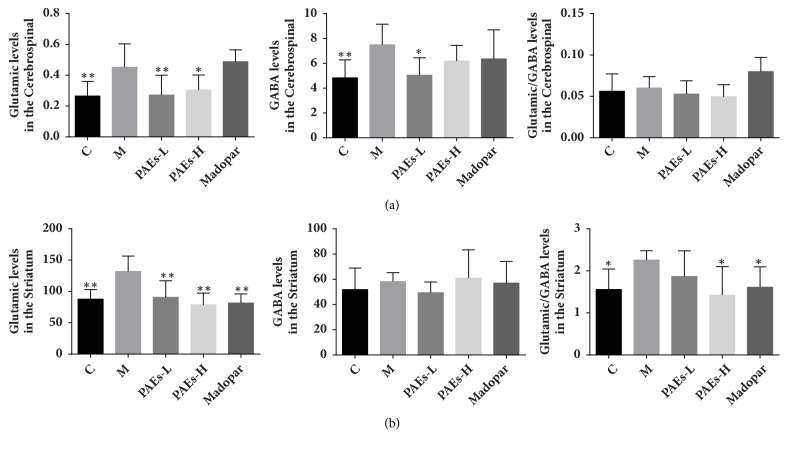
PAEs upregulated the levels of glutamate and GABA in the CSF and striatum of PD rats model. (a) 6-OHDA enhanced remarkably glutamate and GABA in CSF of rats, while 60 mg/kg·d, 180 mg/kg·d PAEs administration restore glutamate and GABA to nomal level, but Madopar had no significantly changed. (b) 6-OHDA increased glutamate and GABA in striatum of rats, while 60 mg/kg·d and 180 mg/kg·d PAEs administration significantly decreased glutamate level (*∗*p<0.05 versus model, *∗∗*p<0.01 versus model).

Compared to the control group, excitatory amino acids glutamate and inhibitory amino acid GABA in the CSF increased remarkably in rats given 6-OHDA. PAEs treatment restored glutamate and GABA to normal level; however Madopar had no significant effect on glutamate and GABA levels (Figure 5(a)). However, higher glutamate levels in the striatum were observed, with no changes to striatum GABA levels. Lower glutamate levels in the striatum were observed in rats given PAEs and Madopar, which indicated increased excitotoxicity in the striatum (Figure 5(b)).

### 3.5. PAEs Protects DA Neurons against 6-OHDA Induced Neurotoxicity

**Figure 6:**
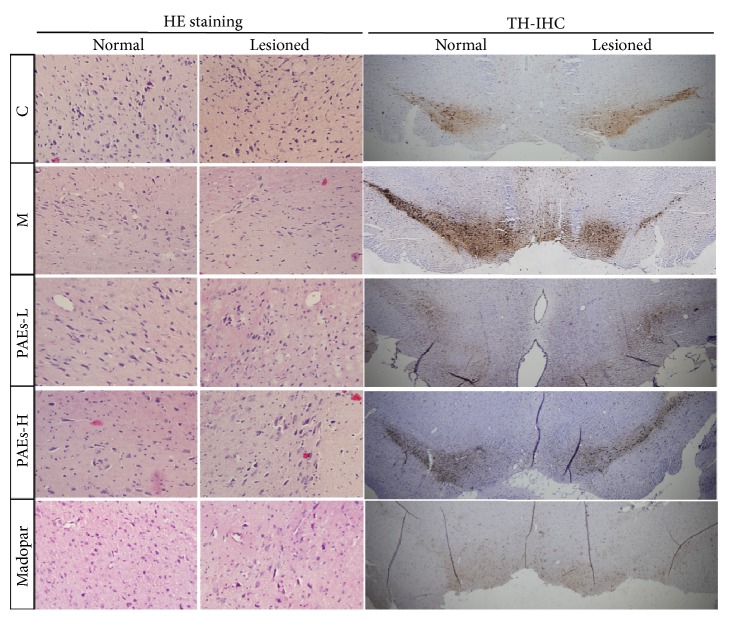
PAEs protect neuronal cells against 6-OHDA induced neurotoxicity. (a) HE staining showed that PAEs inhibited 6-OHDA induced neuronal cell death in the SNc. 6-OHDA administration significantly induced neuronal cell apoptosis. Administration of 60 mg/kg·d and 180 mg/kg·d PAEs inhibited neuronal cell apoptosis induced by 6-OHDA and its inhibition was higher compared to 10 mg/kg·d Madopar administration. (b) TH immunostaining demonstrated that PAEs could inhibit 6-OHDA induced TH-positive cell death in the SNc. TH-positive cells were reduced in 6-OHDA lesions in the SNc. Administration of 60 mg/kg·d and 180 mg/kg·d PAEs inhibited cell death induced by 6-OHDA, and its effect was comparable to 10 mg/kg·d Madopar treatment.

HE staining demonstrated that the number of DAergic neuronal cells on the unaffected side of the brain were ribbon-like arranged, while DAergic neuronal cells on the 6-OHDA lesioned side of SNc were decreased significantly. The number of DAergic neuronal cells was higher in the PAEs and Madopar administered group (Figure 6(a)).

TH immunostaining showed that there was a loss of TH-positive neurons in the SNc after 6-OHDA administration. Higher TH-positive neurons in the SNc were observed in the PAEs and Madopar administered group. The protective effect of PAEs-H in TH-positive neurons was higher compared to the PAEs-L and Madopar group (Figure 6(b)).

### 3.6. Expression of GAP-43 and NF-H in the SNc and Striatum

**Figure 7:**
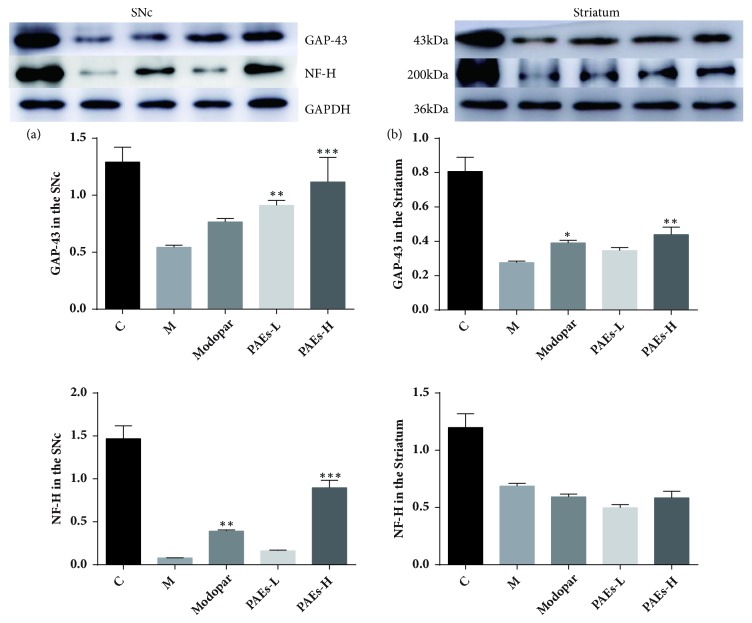
PAEs increase GAP-43 and NF-H expression levels in the SNc and striatum of 6-OHDA induced PD rats. GAP-43 and NF-H expression after 6-OHDA administration was detected in the SNc (a) and striatum (b). The expression of GAP-43 and NF-H in SNc after administration with 60 mg/kg·d and 180 mg/kg·d PAEs (a) and striatum (b) increased remarkably. Expression was comparable to 10 mg/kg·d Madopar, especially in the 180 mg/kg·d PAEs administered rats.

GAP-43 and NF-H protein expression in the SNc and striatum of PD rats were measured by Western Blot. Compared to the model group, after treatment with PAEs and Madopar, GAP-43 levels in the SNc and striatum were higher. Compared to Madopar administered rats, GAP-43 levels were slightly higher in the SNc and striatum in the PAEs group and were dose dependent (Figure 7). NF-H protein expression was also significantly reduced in the model group compared to the control group. Results demonstrated a slight increase in NF-H levels in the SNc of PD rats that received PAEs-L. However, Madopar and PAEs-H significant increased NF-H levels in the SNc (Figure 7). In the striatum of PD rats, NF-H levels were reduced compared to the control group, and no detectable changes were observed after PAEs-L, PAEs-H, or Madopar administration (Figure 7).

### 3.7. Identification of PAEs Components after Intragastric Administration

**Figure 8:**
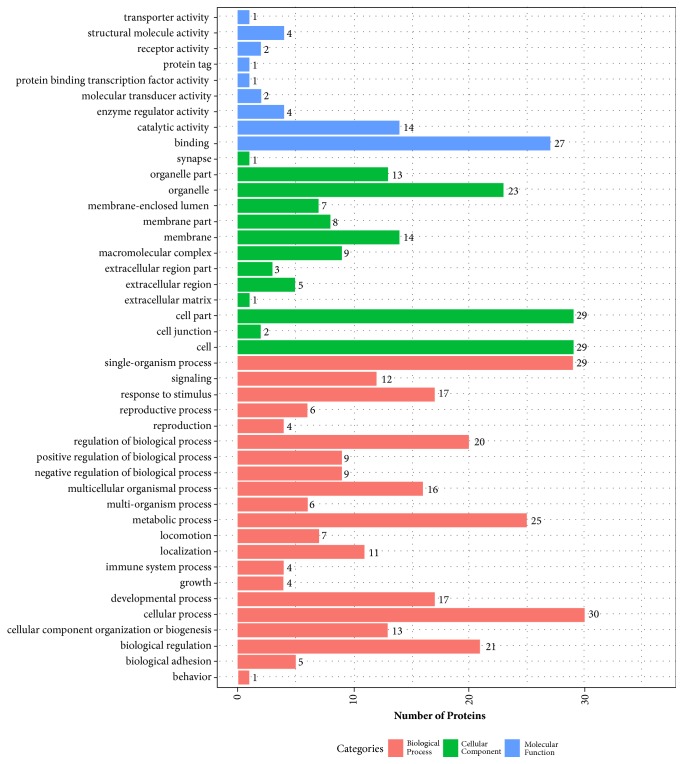
Functional cluster analysis of identified proteins in PAEs. Biological process, cellular component and molecular function of PAEs identified are shown above. 21 biological processes were identified, which included developmental process, growth, reproductive process as well as 13 cellular components, which included membrane-enclosed lumen, macromolecular complex. Proteins were also categorized into 9 molecular functions, which included catalytic activity and structural molecule activity.

Transcriptomic analysis of Pilose antler showed that the total number of proteins expressed in Pilose antler was 92045, including protein without sequence number. In comparison to the number of proteins detected in Pilose antler extracts, 90 proteins were detected in the blood serum of 6-OHDA administered rats. In blood serum of rats administered with PAEs-H, 76 Pilose antler proteins were detected. Total numbers of distinct Pilose antler proteins in the blood serum of rats that were administered with 6-OHDA and PAEs-H were 49, of which the biological processes, cellular components, and molecular functions of these proteins are shown in Figure 8. Among these 49 proteins, 11 Pilose antler proteins had been reported to have neuropharmacological activity, e.g., maintenance of synaptic plasticity or promoting neurite outgrowth (Table 1).

## 4. Discussion

Numerous* in vitro* and* in vivo* studies have demonstrated the pharmacological activity of Pilose antlers in the nervous system. These studies have demonstrated that polypeptides extracted from Pilose antler promoted the differentiation of rat brain-derived neuronal stem cells [19], promoted spinal cord injury repair in rats [20], and facilitated neuronal cell regeneration [21]. Velvet antler polypeptides also had protective effects against MPP^+^-induced SH-SY5Y cell death, which suggests the anti-Parkinsonism activity of velvet antler polypeptides [22]. Although these results highlight the neuropharmacological potential of velvet antlers, the studies were limited to* in vitro* studies or locally administered. This seems at odds with the traditional way TCM is administered. Thus, it was necessary to evaluate the activity of velvet antlers* in vivo* studies.

In this study, the neuroprotective potential of PAEs after 6-OHDA induced Parkinson was evaluated in a rat model. PAEs restored the levels of monoamine neurotransmitters and amino acids in the striatum and cerebrospinal fluid, such as DA, DOPAC, HVA, 5-HT, and their metabolites in 6-OHDA induced rat models. In addition, PAEs decreased the level of Glu and GABA levels. However, the level of monoamine neurotransmitters and amino acids mentioned above only partially reflects the progression of PD. The most characterized pathological feature of PD is considered to be DAergic neuronal cell death, which leads to striatal DA depletion [23]. TH is the rate-limiting enzyme for catecholamine synthesis and loss of nigrostriatal DA directly associates with TH inactivation. Thus, the amount of TH-positive neuronal cell is a critical marker for DAergic neuronal cell. In this study, we found that PAEs inhibited 6-OHDA-induced TH-positive neuronal cell death.

PAEs also increased the levels of GAP-43 and NF-H in rat brain tissue, which suggests that PAEs may facilitate neuronal growth and plasticity. This also indicated a possible mechanism for the protective effect of PAEs against 6-OHDA-induced neuronal cell death and may tightly correlate with the components in PAEs promoting neurogenesis.

Although these results demonstrated the anti-Parkinsonism activity of PAEs, identifying the causative components in PAEs has yet to be deciphered. In our previous study, proteomic analysis with molecular tracing techniques and mass spectrometry identified certain proteins and peptides components in PAEs that cross the gastric mucosa and remained intact from enzymatic proteolysis [24]. This indicated that these proteins or peptides were in the blood circulation and most likely were responsible for neuroprotection. By proteomic analysis, 49 Pilose antler proteins were identified in blood serum of rats that were given PAEs. 11 Pilose antler components identified in the blood serum had been reported to exert pharmacological effect in the nervous system, such as promoting neuronal growth, survival, and regeneration, facilitating neurite outgrowth and plasticity. Small GTPases [9], one of the Pilose antler components found in the blood serum of rats administered with PAEs, is a protein involved in regulating a plethora of cellular functions including synaptic plasticity. Collagens [17] account for over 50% of the components of Pilose antler extracts and have been reported to promote neurite outgrowth. These Pilose antler components could be the ones responsible for the neuroprotective effect of PAEs.

Blood-brain barrier (BBB) penetration or indirect regulation is involved in the therapeutic regulation of compounds in the nervous system. It has been reported that molecules with a molecular weight larger than 450 Da cannot penetrate across the BBB [25]. Further proteomic analysis using BBB models is needed to validate our preliminary findings.

## 5. Conclusions

These results demonstrate that first* in vivo* evidence on PAEs protects against 6-OHDA induced toxic effects in the PD rat models. Therefore, oral administration of PAEs may be a new drug for neurodegenerative disorders like PD.

## Figures and Tables

**Table 1:** Pilose antler proteins having neuropharmacological activity.

**Protein**	**Function**
Dystonin	a mediator of normal endoplasmic reticulum structure and function [8]
Small GTPases	synaptic plasticity [9]
Beta amyloid precursor protein	synaptic plasticity [10]
lamin	motor neuron regeneration [11]
Profilin	neurite outgrowth [12]
TNF receptor-associated factor	Anti-inflammatory [13]
Integrin	embryonic development, muscle cell adhesion and contraction, and migration of nerve cell axons and gonadal distal tip cells [14]
Cystathionine beta-synthase	Anti-oxidation [15]
Alpha-macroglobulin	inhibits 6-OHDA-induced oxidase stress injury [16]
Collagens (type IV and type XIII)	neurite outgrowth [17]
Talin	Axon Growth and Regeneration [18]

## Data Availability

The data used to support the findings of this study are available from the corresponding author upon request.
